# Ageing-induced shrinkage of intervessel pit membranes in xylem of *Clematis vitalba* modifies its mechanical properties as revealed by atomic force microscopy

**DOI:** 10.3389/fpls.2023.1002711

**Published:** 2023-01-23

**Authors:** Cora F. Carmesin, Fabian Port, Samuel Böhringer, Kay-Eberhard Gottschalk, Volker Rasche, Steven Jansen

**Affiliations:** ^1^ Institute of Systematic Botany and Ecology, Ulm University, Albert-Einstein-Allee 11, Ulm, Germany; ^2^ Institute of Experimental Physics, Ulm University, Albert Einstein Allee 45, Ulm, Germany; ^3^ Institut für Quantenphysik and Center for Integrated Quantum Science and Technology, Universität Ulm, Albert-Einstein-Allee 11, Ulm, Germany; ^4^ Core Facility Small Animal Imaging, Medical Faculty, Ulm University, Ulm, Germany; ^5^ Department of Internal Medicine II, Ulm University, Albert Einstein Allee 45, Ulm, Germany

**Keywords:** AFM, angiosperm xylem, bordered pit, mechanobiology, pit membrane, Qi, wood, elastic modulus

## Abstract

Bordered pit membranes of angiosperm xylem are anisotropic, mesoporous media between neighbouring conduits, with a key role in long distance water transport. Yet, their mechanical properties are poorly understood. Here, we aim to quantify the stiffness of intervessel pit membranes over various growing seasons. By applying an AFM-based indentation technique “Quantitative Imaging” we measured the effective elastic modulus (*E* ^effective^) of intervessel pit membranes of *Clematis vitalba* in dependence of size, age, and hydration state. The indentation-deformation behaviour was analysed with a non-linear membrane model, and paired with magnetic resonance imaging to visualise sap-filled and embolised vessels, while geometrical data of bordered pits were obtained using electron microscopy. *E* ^effective^ was transformed to the geometrically independent apparent elastic modulus *E* ^apparent^ and to aspiration pressure *P*_b_. The material stiffness (*E* ^apparent^) of fresh pit membranes was with 57 MPa considerably lower than previously suggested. The estimated pressure for pit membrane aspiration was 2.20+28 MPa. Pit membranes from older growth rings were shrunken, had a higher material stiffness and a lower aspiration pressure than current year ones, suggesting an irreversible, mechanical ageing process. This study provides an experimental-stiffness analysis of hydrated intervessel pit membranes in their native state. The estimated aspiration pressure suggests that membranes are not deflected under normal field conditions. Although absolute values should be interpreted carefully, our data suggest that pit membrane shrinkage implies increasing material stiffness, and highlight the dynamic changes of pit membrane mechanics and their complex, functional behaviour for fluid transport.

## Introduction

### Angiosperm pit membranes: Anatomy, ontogeny, functions and potential trade-offs

Interconduit pit membranes in bordered pit pairs represent important structures for the long distance water transport in xylem tissue of vascular plants (Kenrick and Crane, 1997; Pittermann, 2010). Pits are openings in the secondary cell wall between neighbouring conduits, and show a characteristic pit border with overhanging secondary walls in water-conducting tracheids and vessels. During its development, the primary cell wall and middle lamella undergo structural and chemical modifications, resulting in a pit membrane as physical barrier between neighbouring conduits (Schmid & Machado, 1968). The pit border forms a fairly enclosed cavity, and is assumed to provide mechanical support to the pit membrane in case it would be deflected (i.e. aspirated) (Carlquist, 2001; Kaack et al., 2019). Mature pit membranes are mainly composed of cellulose microfibril aggregates. These structures are defined as grouped cellulose fibrils, which aggregate into variable sizes of a few to many cellulose fibrils (Kaack et al., 2019). They have a thickness between ca. 200 and 1,200 nm (Li et al., 2016; Kaack et al., 2019; Kaack et al., 2021) and were found to represent mesoporous media. Despite a considerable variation in pit membrane thickness (Choat et al., 2003; Zhang et al., 2020; Kaack et al., 2021), pore constriction sizes in angiosperm pit membranes are within the range of 2 to 50 nm.

The pore constriction size is important for our understanding of fluid (liquid and gas) transport across these physical barriers (Kaack et al., 2019), and how pit membranes may or may not represent a possible trade-off between hydraulic safety and efficiency (Kaack et al., 2021). On the one hand, pore constriction sizes in pit membranes need to be large enough to provide a high permeability and low hydraulic resistance to sap flow, but on the other hand, pit membranes should prevent embolism spread from an embolised to a sap-filled conduit (Avila et al., 2022). In a relaxed state and under moderate flow conditions, the number and the size of pore constrictions is highly determined by the pit membrane thickness, although thickness in fresh membranes does not seem to affect the pore volume fraction, which is typically around 80% (Zhang et al., 2020).

In addition to geometric parameters of bordered pits, the elasticity of pit membranes is assumed to play a functional role in responding to changing pressure differences between adjacent conduits. It has been speculated, for instance, that the elasticity of pit membranes may determine whether embolism propagation (traditionally described as “air-seeding”) occurs *via* reversible or permanent enlargement of pore constrictions (i.e., capillary failure after pit membrane deformation), or through irreversible pit membrane rupture (Choat et al., 2004; Sperry and Hacke, 2004; Choat et al., 2008; Zimmermann, 1983). Therefore, potential trade-offs in a plant’s life history, growth rate, and longevity (Roskilly et al., 2019), could also be reflected in the functional anatomy of conduits, with apparent trade-offs between functional demands, such as efficient water transport to promote growth, and mechanical safety to impede embolism (Zimmermann, 1983; Baas et al., 2004; Chave et al., 2009; Gleason et al., 2016).

### Why investigating the mechanical properties of hydrated angiosperm pit membranes is demanding, but relevant

While few studies investigated the mechanical properties of torus-margo pit membranes in gymnosperms (Schulte et al., 2015; Zelinka et al., 2015; Schulte and Hacke, 2021), experimental studies on the mechanical properties of homogeneous pit membranes in angiosperms are very limited (Capron et al., 2014; Tixier et al., 2014). In general, proven high-resolution imaging techniques require dry and/or chemically modified material and thus cannot be used for mechanical elucidation. While gymnosperm pits allow for technically less demanding elasticity measurement methods due to their relatively large dimensions (Zelinka et al., 2015), the application of nano-indentation techniques to angiosperm pit membranes is technically challenging due to the highly structured nature of xylem and potential artefacts by dehydration and shrinkage. However, there is a need to investigate the ultrastructure and mechanical properties of pit membranes in their native, never-dried state (Pesacreta et al., 2005) because intervessel pit membranes have been shown to undergo considerable and largely irreversible shrinkage during dehydration (Zhang et al., 2020). We are interested to know how both the original and changing mechanical properties determine their reaction to mechanical loads and affect fluid transport.

### Continuous and occasional mechanical loads on pit membranes

The inner layers of a pit membranes are formed before cell expansion has finished, while outer layers are deposited after its final size has been reached. So, at least the inner layers are under pre-stress (Schmid and Machado, 1968). Additionally, conduit cell walls are subject to a peripheral stress caused by a relatively wide range of xylem sap pressures, from close to zero to negative values well below -1 MPa (Hacke et al., 2001; Choat et al., 2012). A locally homogeneous negative pressure would put an interconduit pit membrane permanently under symmetric external load, while a very low but additional, asymmetric force is expected under conditions of unidirectional flow. While some studies focussed on flow simulations of angiosperm pit membranes and estimations of the hydraulic resistance, almost nothing is known about potential fatigue phenomena, or how flow may affect the ultrastructure of pit membranes (Xu et al., 2012; Park et al., 2019; Park et al., 2021). Strong pressure differences at pit membranes have been suggested to occur between an embolised conduit and a neighbouring, sap-filled one. These pressure differences may provoke deflection and deformation of pit membranes, which may increase the risk of gas spreading to an intact vessel by direct rupture of the pit membrane and/or enlargement of pore constrictions (Sperry & Hacke, 2004; Choat et al., 2004).

Pit membrane thickness itself is determined not only by the number of microfibrillar layers of cellulose, but also by the hydration state, the intercellulose hydrogen bonds, and cellulose-water hydrogen bonds. Dehydration has found to cause shrinkage in membrane thickness and is suggested to exert strong forces. Since pit membrane shrinkage is associated with a major reduction of the porosity (i.e., the pore volume fraction; Zhang et al., 2020) it is possible that shrunken, compact pit membranes show a different elasticity than non-shrunken ones. While rehydration of a dried pit membrane did not show any difference in elasticity of pit membranes in *Populus* (Capron et al., 2014), the elasticity of pit membranes in fresh samples has not been analysed yet quantitatively according to the available literature. Moreover, the irreversible nature of pit membrane shrinkage is not fully understood, but could be caused by the formation of strong hydrogen bonds between cellulose fibrillar aggregates (Zhang et al., 2020). Shrinkage of intervessel pit membranes could be artificially induced by dehydration in the lab (Zhang et al., 2017; Kotowska et al., 2020), but is also known to occur under natural conditions in the field (Jansen et al., 2018), with a 50% shrinkage across a single growing season in grapevine (Sorek et al., 2021).

As such, pit membranes are compliant, porous media with a non-negligible deformation potential, similar to many other porous media in nature in which deformability may depend on the porous medium matrix, the fluid pressure, and/or the flow conditions (e.g., slow vs rigorous flow) (Wang, 2000; Coussy, 2004; Cheng, 2016). Whether or not cellulose fibrillar aggregates in pit membranes can easily re-arrange upon flexing or stretching is unknown, and may depend on the distance from the pit membrane annulus, where the cellulose bundles are anchored into a pectin-rich ending of the primary wall.

### Our study

We established an AFM-based method to obtain several mechanical parameters of pit membranes by nano-indentation in (1) a close-to-native, hydrated state, and a (2) rehydrated state and tested whether they were affected by the following three parameters: pit membrane size, age, and hydration state. We expected a significant stiffening, and an irreversible stiffening increase in shrunken pit membranes compared to fresh ones. It is likely that fresh and dehydrated pit membranes show not only structural but also mechanical differences due to dehydration-induced intercellulose hydrogen bonds (Chen et al., 2018; Li et al., 2020), irreversible shrinkage of pit membranes (Kotowska et al., 2020; Zhang et al., 2020), or other ultrastructural changes, such as re-arrangement of cellulose microfibril aggregates (Pesacreta et al., 2005). Therefore, not only dehydrated, but also rehydrated pit membranes should show different mechanical properties. As far as we know, the only quantitative experimental study to date has been performed on intervessel pit membranes of *Populus deltoides* x *Populus nigra*, but showed no difference in the stiffness between dried and rehydrated pit membranes (Capron et al., 2014). This non-intuitive result shows once more the need for further studies to elucidate intrinsic pit-membrane properties with the aim to predict their mechanical response to different loads. Since the number of AFM studies on fresh pit membranes is very limited, we also include detailed, descriptive information based on high-resolution AFM imaging, which we combine with field-emission SEM. The overall goal of our study was to contribute to the understanding of how the original stiffness of fresh membranes determines their behaviour (bending, pore size stretching) under mechanical loads (flow-induced pressure differences, embolism-induced pressure differences) and how the stiffness itself is influenced by dehydration-induced shrinkage. All features are likely to influence fluid transport efficiency through the membrane, and are therefore relevant at the organismic level.

## Material and methods

### Plant material

We studied early-wood vessels of the European liana species *Clematis vitalba* L., which is characterized by relatively large conduits with easily exposable intervessel pit membranes. Samples were taken from eight individuals growing near Ulm University and Örlinger Tal (Germany, 48°25’20.3” N, 9°57’20.2” E). Internodal segments of about 10 cm long were cut off from stems, which were at least six-years old, and immediately submersed in a commercial mineral water (Volvic; minerals included: Ca^2+:^ 12 mg/l; Mg^2+^: 8 mg/l; Na^+^: 12 mg/l; K^+^:6 mg/l; Cl^-^:15 mg/l; $\text{SO}4_{2}^{-}$ : 9 mg/l; HCO^3-^: 74 mg/l; SiO_2_: 32 mg/l; pH = 7.00) to avoid dehydration. By using commercial mineralized water, we wanted to keep a controlled ion composition, as an ionic effect on pit membrane structure is known (Lee et al., 2012). Furthermore, we wanted to keep the physiological state of the samples as close as possible to the original state within the plant.

### Preparation of exposed intervessel pit membranes for scanning electron microscopy and atomic force microscoy

We aimed to obtain wood samples with large intervessel pit fields and exposed intervessel pit membranes, which were not covered up by secondary cell wall. Although vessels have different pit types, such as vessel-parenchyma, vessel-tracheid, and intervessel pit membranes, we used the term pit membrane in this paper as a synonym for intervessel pit membrane. In the lab, the bark was first peeled off from stem segments. Cross sections were made and then observed to detect paired vessels under a stereo-microscope. If this was the case, the sample was longitudinally split between two paired vessels, and further trimmed if needed. This was done with a razor blade, tweezers, and needles, and special care was taken to keep the samples fully hydrated all the time during sample preparation. As a result, we obtained two mirroring samples, with dimensions of approximately 2 x 0.2 x 1mm (length x width x height).

In winter, trimming of the specimens caused a wound response within a few minutes, which was characterised by the secretion of a viscous fluid, affecting the AFM measurements by changing the viscosity of the medium and by covering the sample. Therefore, the samples had to be processed quickly, and were washed with Volvic water several times before AFM was applied. Once processed and washed, the secretion was no longer observed.

We prepared fresh, never-dried samples of pit membranes from the current year (age = 0), and samples of the secondary xylem that were one to four years old. Samples that were successfully measured by AFM were carefully dehydrated over several days in chambers with a decreasing relative humidity (100%, 75%, 33%, 23%), gradually or immediately rehydrated, and then scanned again by AFM to determine the effect of dehydration on pit membrane stiffness.

### Scanning electron microscopy

Samples gained by following the protocol described above were air-dried at room temperature, gold-coated (approx. 15 nm) with a Sputter Coater (108 auto/SE, Cressington Scientific Instruments Ltd., Watford, UK), and analysed with a Hitachi cold field-emission SEM S-4700 (Hitachi high technologyies Corp., Tokyo, Japan) at about 2kV.

### Atomic force microscopy

#### General procedures for sample preparation

We prepared wood samples of different ages and hydration state as described above, and fixed the two mirroring samples in a clamp (**Appendix S 1**) with the fracture surfaces between two paired vessels being orientated parallel to the ground plane. The clamp was in-house designed for AFM-measurements in a liquid environment. The clamped samples were stored in Volvic water in a fridge until measured in the same medium.

#### High-resolution imaging based on AFM in the tapping mode

Atomic force microscopy was carried out within less than 24 hours after sampling. High-resolution AFM images of the pit membranes were obtained at room temperature in a Volvic medium with a NanoWizard 3 Ultra (JPK BioAFM, Bruker Nano GmbH, Berlin) mounted on an inverted optical microscope (Axio Zoom.V16, Carl Zeiss, Oberkochen, Germany) using the tapping mode. We used an Olympus micro cantilever OMCLAC240TSA-R3E with gold coating (spring constant 2 N/m, resonant frequency 70 kHz).

Before measurements were performed, the cantilever was submersed in the Volvic water and left there for 10 min. Deflection sensitivity and spring constant of the cantilever were calibrated with a contactless calibration method from the manufacturer JPK, so no substrate was needed. We required the cantilever dimensions, which were provided by the AFM-tip distributor.

For overview pictures, an area of 20 x 20 µm with 128 x 128 pixels was chosen. For an image-filling scan of a pit membrane we choose 6 x 6 µm with 512 x 512 pixels. For high resolution measurements, we scanned membrane areas of 1 µm x 1 µm with 512 x 512 pixels.

#### Mechanical mapping using Quantitative imaging mode

Force-distance curves were collected at room temperature in Volvic water with a NanoWizard 3 Ultra (JPK BioAFM, Bruker Nano GmbH, Berlin) mounted on an optical microscope (Axio Zoom.V16, Carl Zeiss, Oberkochen, Germany) using the “Quantitative Imaging (QI™)” measuring mode. We used a cantilever with a spherical tip, with a radius of 500 nm, a nominal spring constant of 0.2 N/m, and gold reflective coating (biosphere B500-CONT, NanoAndMore GmbH, Wetzlar, Germany). The same calibration was applied to the two types of AFM-tips used in this study.

We collected overview pictures of 20 x 20 µm and 32 x 32 pixels (**Appendix S 2a**) and more detailed pit membrane pictures at 10 x 10 µm and 20 x 20 pixels (**Appendix S 2b**). The same basic settings were kept for all scans: constant speed mode; z-length = 3000 nm; set point: 0.6-1.3 nN, depending on the sample; extend time = 300 ms; extend speed = 15 µm/s; extend sample rate = 100 kHz; retract time = 300 ms; retract sample rate = 100 kHz; motion time = 6.0 ms; acceleration = 1.0 ms; next line time = 1200.0 ms; next line delay = 500.0 ms; next line retract = 900.0 nm.

### Transmission electron microscopy

TEM was applied to evaluate pit membrane thickness, following a standard protocol (Jansen et al., 2009, Scholz et al., 2013; Li et al., 2016). Blocks of about 1 to 2 mm^3^ were cut from the current growth ring, and wood from growth rings that were between two years and five years old. Samples were treated with a standard fixative (2.5% glutaraldehyde, 0.2 mol phosphate, 1% sucrose, pH 7.3, 500 µl). Afterwards, the samples were washed in a 0.2 M phosphate buffer, and postfixed with 2% buffered osmium tetroxide for 2 to 4 hours at room temperature. The samples were then washed with a buffer solution, and gradually dehydrated in a propanol series (30%, 50%, 70%, 90%, 100%). Samples were then embedded in Epon resin (Sigma-Aldrich, Steinheim, Germany). Transverse, semi-thin sections (500 µm) were cut from the embedded samples with a glass knife, stained with 0.5% toluidine blue and mounted on slides. Ultrathin cuts (70 to 100 nm) were made with a diamond knife and placed on Formvar grids. The samples were analysed with a JEOL 1400 TEM (JEOL, Tokyo, Japan) at 120 kV. TEM fixation and dehydration appears not to lead to major changes in pit membrane thickness based on a comparison of various methods (SJ; unpublished data).

### Magnetic resonance imaging

Magnetic resonance imaging (MRI) allowed us to visualise the sap-filled and gas-filled xylem conduits in plants *in situ* (Holbrook et al., 2001; Kaufmann et al., 2009; Choat et al., 2010; Choat et al., 2016).

To visualise the sap-filled conduits in *Clematis vitalba*, an intact 3-year old plant grown outside in the Botanical garden of Ulm University was dug up with roots, transferred to a pot, and pulled with the entire stem through a high-field MRI (BioSpec 117/16, Bruker Biospin) at the Core Facility Small Animal Imaging of Ulm University. The measurement was done one week after potting, so that the plant had sufficient time to adapt to the potted conditions. Since the leaves of the plant remained fully turgescent after potting, we assumed that the plants were functioning normally after one week, and that no artificial embolism was induced in the stem section that was scanned with the MRI instrument. The plant had a total stem length of ca. 5 meter, and the area scanned was at ca. 1.5 m from the stem apex.

The stem was fixed in place using tape and scanned repeatedly between 26 and 30 May 2011, i.e. after full leaf expansion and development of new functional vessels. The plant was well watered with tap water. All MRI data were acquired with a high-resolution multi-slice Rapid Acquisition with Relaxation Enhancement (RARE) sequence with acquisition parameters as: spatial resolution Δ*r* = 34 x 43 x 500 µm³, echo/repetition time TE/TR = 30 ms/1150 ms, pixel bandwidth bw = 50 Hz. After scanning the intact plant stem, we cut out a small stem segment, which was flushed with tap water, and then performed a similar scan.

### Anatomical and physical parameters measured

**Figure 1:**
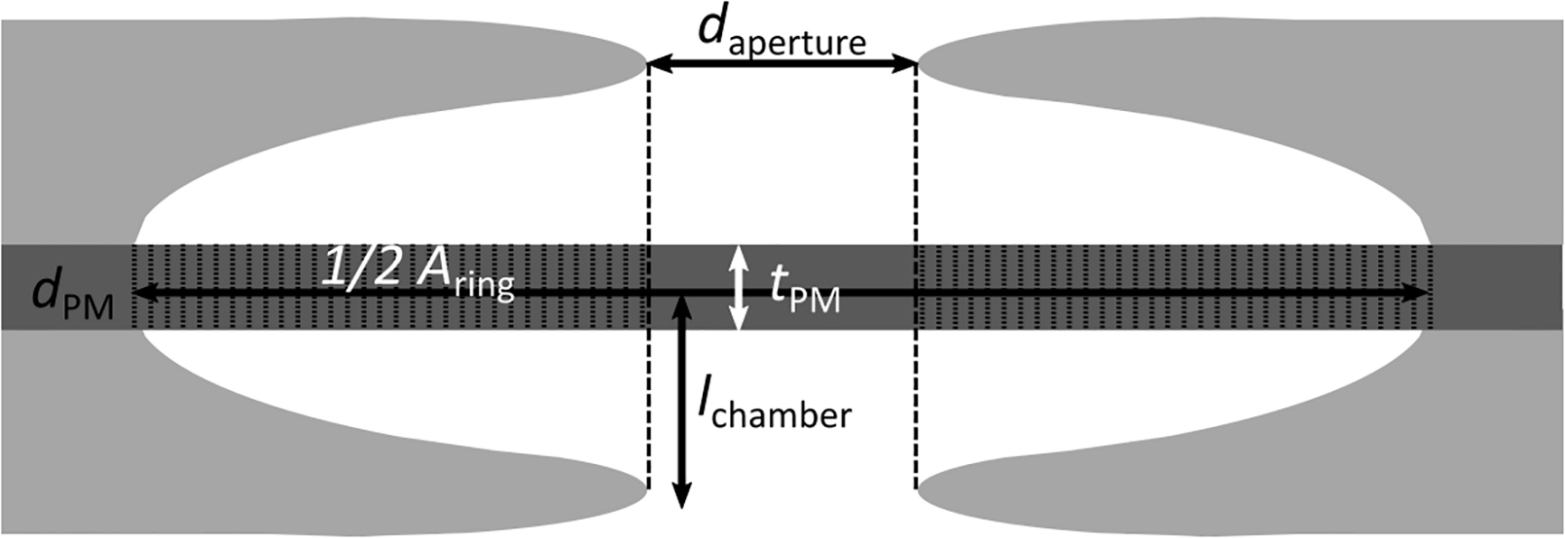
Anatomical parameters measured in pit membranes, visualized in a median transverse section through a single pit. *d*_aperture_: Diameter of the aperture, measured in SEM pictures. *d*_PM_: diameter of the pit membrane, measured in AFM and SEM pictures. *A*_ring_: Area of the pit membrane not covered by the projection of the aperture, calculated based on *d*_aperture_ and *d*_PM_. *t*_PM_: thickness of the central pit membrane, measured in TEM pictures. *l*_chamber_: chamber depth, measured in TEM pictures.

In addition to qualitative pit membrane features based on SEM, we measured several parameters quantitatively (**Figure 1**). Normally distributed data were given as mean and standard deviation (SD) and were subjected to parametric tests. Non-normally distributed data were presented as median values with their range, unless we wanted to compare it with normally distributed data, and subjected to non-parametric tests.

#### Anatomical parameters measured

We measured the diameter of cellulose-microfibril aggregates (*d*_cellulose_) in a fresh, never-dried pit membranes based on a single, high-resolution picture taken in AFM tapping mode using ImageJ V 1.50e (Wayne Rasband, National Institutes of Health, USA) and repeated the measurements for a dried pit membrane based on a single, high-resolution SEM picture.

At the pit membrane level (**Figure 1**), we measured the diameter of the membrane (*d*_PM_) using the same AFM-QI-images taken for the elasticity measurements and evaluated them with the data processing software provided by the manufacturer (JPK BioAFM, Bruker Nano GmbH, Berlin). For each membrane, the diameter was measured along the two orthogonal main axes, because the pit membranes were not perfectly circular. The average value of both diameters was taken as pit diameter. Additionally, we repeated diameter measurements also for SEM samples using ImageJ V 1.50e (Wayne Rasband, National Institutes of Health, USA). In the same way, we measured the diameter of the outer pit aperture (*d*_aperture_) to calculate the area (*A*_ring_) of the pit membrane that would be supported by the pit border in case the membrane would be aspirated, i.e. fully deflected until it would hit the roof of the pit chamber by assuming a 2D-projection (Equ. (1).


(1) $$A_{\text{ring}}=\frac{\pi }{4}\;(d_{\text{PM}}^{2}-d_{\text{aperture}}^{2})$$

*A*_ring_ was needed to estimate the minimum pressure that was required to aspirate the pit membrane. The average diameter was taken for both the pit membrane and the outer pit aperture. Normal distribution of *A*_PM_ and *A*_ring_ was tested based on a Shapiro-Wilk-Test. The correlation between *A*_ring_ and *A*_PM_ was then determined with Spearman’s Test for not normally distributed data.

The interevessel pit membrane thickness (*t*_PM_) of three *Clematis vitalba* individuals was measured 2019 and 2021 in four different growth-rings (age=0 (current year), 1, 2 and 4 years), corresponding to xylem with a different age. To distinguish between age and ageing effects in thickness, we sampled the same individual in both years, so that growth ring of age = 0 in 2019 corresponded to growth ring of age = 2 (2021).

All measurements were based on TEM samples using ImageJ V 1.50e (Wayne Rasband, National Institutes of Health, USA). The thickness measurements were taken in the centre of the pit membrane, with three measurements per pit membrane (pit number *n*_age=0 =_ 11; *n*_age=1 =_ 5; *n*_age=2 =_ 2; *n*_age=4 =_ 20). Then, the average value was calculated. So far, no comparative data of thickness measurements according to different preparation methods have been published. However, the available evidence supports the hypothesis that TEM fixation and dehydration does not lead to major changes in pit membrane thickness (SJ; unpublished data).

The pit chamber depth (*l*_chamber_) was highest in the centre, but typically lower near the pit membrane border in case sections were not cut through the centre. Therefore, the chamber depth *l*_chamber_ was calculated on TEM images of pits that showed two opposite pit apertures within a bordered pit pair. The entire depth of the bordered pit pair was measured (i.e. from one pit border to the neighbouring one), and then divided by two to have the chamber depth of a single pit border. Then, the average value was calculated (pit number *n* =26).

The ratio between the depth of the pit chamber to the radius of the pit membrane (*l:r*) was calculated using the average values.

#### Physical properties of pit membranes

##### Effective stiffness modulus *E* ^effective^ and apparent elastic modulus *E* ^apparent^ *via* pit membrane modelling based on a Hertz model and a lipid-bilayer model

When condensed matter is exposed to external loads, the material as a consequence will deform and/or deflect. The relative change of the material in length (1D), area (2D) or volume (3D) is called strain. This strain in turn causes internal stresses of the study material. In an equilibrium state, forces applied by internal stresses act in an opposite direction to the given external loads and compensate for them. For strains within the elastic regime, stress is a linear function of strain, with an elastic modulus as the proportionality constant. The apparent elastic modulus *E* with Pa being Pascal) evaluated here is a intrinsic material property, quantifying stiffness of the non-isotropic membrane, whose elastic response includes stretching and bending components. We therefore do not apply the more clearly connoted term Youngs’ modulus, which has been used synonymously in other publications. However, both moduli behave in a similar way: the higher the value, the stiffer the material is.

The protocol of stepwise data modelling was preceded by the fact that it produced a parameter as an intermediate step, which we called effective stiffness modulus (*E* ^effective^) with $[E^{\text{effective}}]=\frac{Pa}{m}$ m being meter). *E* ^effective^ is a mechanical property of a pit membrane as a discreet object and reflects the force required to deflect the system. Thus, it might be relevant for a biological interpretation of our measurements.

We applied a Hertz model and a lipid-bilayer model within a particular order (Hertz fit → baseline correction → Hertz fit →Lipid bilayer model; for more details see **Table 1**) to estimate the effective stiffness modulus and the apparent elastic modulus of pit membranes.

**Table 1:** Overview over the AFM QI data fitting steps to get the parameters Effective stiffness modulus and Apparent elastic modulus.

Step	What applied	Output	Why used	Used data
1	Hertz model	Point of contact, altitude profile	Calibration	Smoothed raw data
2	Base line correction		To eliminate water resistance	Smoothed raw data
3	Hertz model	Indentation depth, penetration depth	Only valid for edges of the membrane, but sufficient to determine the contact point everywhere	Smoothed raw data
4	Plane fit	Tilt correction	Coordinate correction	Contact points
5	Membrane integral	Central position	Estimate centre of membrane, because the following model is only valid in the centre of the membrane	Contact points
6	Bilayer lipid model	Effective stiffness modulus and apparent Elastic modulus	In contrast to hertz model full membrane model	Smoothed raw data
7	Data filter	Negative values excluded, penetration excluded	Sorting for suitable measurements	Smoothed raw data, fit values (E, contact point)
8	GOF filter	Sorting for suitable measurements	Sorting for suitable measurements	GOF

First, the AFM-QI raw data (qi-data.jpk) were calibrated and imported in Matlab with a home-written programme (available upon request). The data were filtered for force-distance curves of more than 700 measurement points to exclude erroneous measurements. The data were then always smoothed with a Gaussian filter. These two steps were repeated prior to each fitting.

To estimate the apparent elastic modulus, we assumed that pit membranes fulfil the following conditions: (1) The membranes were clamped, i.e. the edges are fixed into the primary cell walls *via* the pit annulus, (2) The membranes were circular plates, (3) The membranes were of finite thickness, (4) The pit membranes were loaded perpendicular to the plane during the measurements, and (5) Indentation in the centre caused mainly stretching and subordinated bending.

For the following process we used trace curves only. First, we estimated the point of contact to get the altitude profile from the membrane in the initial state approximating the force-distance curves (Equ. (2) with the Hertz model (Hertz, 1882)):


(2) $$F(z)=\underset{after\;reaching\;the\;contact\;point:\;Hertz\;model}{\underset{︸}{\frac{4}{3}\frac{E}{1-\nu^{2}}R_{t}^{1/2}{\left(\text{z}-z_{0}\right)}^{3/2}\Theta (\text{z}-z_{0})}}+\underset{linear\;relation\;before\;reaching\;the\;contact\;point}{\underset{︸}{\left(-a(z_{0}-\text{z})+f\right)\Theta (z_{0}-\text{z})}}$$

with *F*(*z*) eing the force applied, *z* eing the indentation depth, *E* the elastic modulus, v eiing the Poisson ratio (here 0.5), *R*_*t*_ eing the radius of the tip, *z*_0_ eeing the initial position of the membrane = contact point, θ eing the Heaviside step function, *a* eing the linear calibration coefficient, and *f* eing the force offset.

This was sufficient to calibrate the data by subtracting the baseline in the following step:


$$F^{*}\left(z\right)=F\left(z\right)+a\left(z_{0}-z\right)-f$$

These new force-indentation curves with a corrected baseline were then fitted with the Hertz model again. Thereby, we required the contact point to be close to the initial contact point z_0_ the first fitting, with a range of 20% of the total measured approach.

The above analysis was performed at every pixel per membrane for all membranes, and provided approximate height profiles (**Appendix S 3a**) and penetration depths (**Appendix S 3b**), with the penetration depth being the distance from the point of contact to the maximum force.

A post-processing data analysis followed. We neglected in our force-distance curves points where the pit membrane was penetrated by the AFM tip or disturbed. These were identified by one ore multiple dominant peaks (higher than 0.3 times the maximum force applied) in the smoothed data. If a peak was detected, the data set was excluded. We also excluded points with no or negative slopes, i.e. when the distance between the contact point and the position at maximum force was negative or zero. These data were removed from our analyses. Data filtering was illustrated in **Appendix S 3c**.

Since the measurement plane was tilted with respect to the cantilever axis, this effect has to be systematically corrected for. For this, we fitted a plane through the extremal corner values of each recorded data set, and removed the tilt by subtracting the offset through the cantilever plane.

Before extracting the elastic data with an appropriate model, the central position of the membrane *x_c_,y_c_* ()as estimated based on the indentation profile. We assumed that indentation of the pit membrane was highest in the centre of the pit membrane. Therefore, we evaluated each x-y-position by its normalised indentation depth


$$I_{N}\left(x,y\right)$$

For the x position, we can write


$$(3)x_{c}=\overset{0}{\underset{x_{max}}{\int^{}}}x\;\overset{0}{\underset{y_{max}}{\int^{}}}I_{N}\left(x,y\right)\;dx\;dy$$

with


$$(4)I_{N}\left(x,y\right)=I\left(x,y\right)/\left(\int_{0}^{x_{max}}\int_{0}^{y_{max}}I\left(x,y\right)dxdy\right)$$

*y*_*c*_ as calculated in an analogous way. This procedure is very similar to finding the centre of gravity of a geometric body.

We applied this approach to identify four pixels in the centre of the pit membrane (**Appendix S 3d**).

We performed force-distance fits for the centre of the membrane using smoothed data and a model with nonlinear deformation by stretching only. This model was valid for thin pit membranes with deep indentation (Begley and Mackin, 2004; Janshoff and Steinem, 2015).


$$F\left(z\right)=g\left(\nu \right)\frac{E\cdot t_{\text{PM}}}{R^{2}}z^{3}\Theta \left(z-z_{0}\right)+\left(-a\left(z_{0}{-}_{z}\right)+f\right)\Theta \left(z_{0}-\text{z}\right)$$

th  *F* eing the force applied , *z* eing the indentation depth , *g*(*ν*) ing the numerical solution for all possible Poisson’s ratios =1.05−0.15*ν*−0.16*ν* ^2 ^, *ν* eing the Poisson ratio (here 0.5 , *E*) ing the Apparent elastic modulus, *t*_PM_ eing the thickness of the pit membrane, *R* eing the Radius of the circular membrane  *θ*, ing the Heaviside step function,. *z*_0_ beeing the initial position of the membrane = contact point, θ eing the step function, *a* eing the linear calibration coefficient, an *f* eing the force offset.

Similar to the Hertz model, we needed to fit twice, with a calibration in between.

We allowed the point of contact to vary in a range of 20% from the contact point estimated by the Hertz model. The data were calibrated, subtracting the linear part. For each curve, only 5% of the baseline was included into the model to get better fit results for the relevant part of the curve after contact was made with the pit membrane. Additionally, the goodness of fit was mainly relevant in that part of the curve. Using this approach, we obtained a better measure for the goodness of the non-linear model, which only described the force-distance curve after contact was made with the membrane.

The data were fitted again with


$$F^{*}\left(z\right)=g\left(\nu \right)\frac{Et}{R^{2}}z^{3}\Theta \left(z-z_{0}\right)$$

post-filtered the data by the goodness of the fit, and excluded data with RMSE< 0.7e10-4 (**Appendix S 3e**). In this way, we obtained the Effective elasticity modulus


$$E^{\text{effective}}$$

with


$$[E^{\text{effective}}]=\frac{Pa}{\text{m}}$$

or the four central curves (**Appendix S 3f–i**).


$$(5)E^{\text{effective}}=g\left(\nu \right)\frac{Et}{R^{2}}$$

To obtain the final apparent elastic modulus, we solved Equation (5) for *E* with [*E*]=Pa Therefore, we used the respective radius data for each membrane. For fresh pit membranes, we used the thickness values obtained by TEM data. For artificially dehydrated and rehydrated membranes, we had no thickness data. So, we assumed, that dehydrated pit membranes and fresh 4-year old pit membranes show largely the same thickness. This assumption might be justified based on earlier work (Zhang et al., 2017; Jansen et al., 2018; Kaack et al., 2019; Zhang et al., 2020; Kotowska et al., 2020).

To check if the membrane was lying in a perfect plane, we fitted a plane to the contact points, and then subtracted the plane to get a better height profile of the membrane. For plot generation, we extrapolated the filtered data, and used a smoothing of factor 3.

Finally, we plotted the effective stiffness based on the linear model to get an estimation of the membrane stiffness relative to the stiffness of the surrounding cell wall (**Appendix S 3j**).

##### Pressure difference needed to aspirate the pit membrane

The pressure difference (*P*_b_) required to deflect a fresh pit membrane, developed in the current year, against the pit border was calculated following Capron et al. (2014) and Tixier et al. (2014).


$$(6)P_{\text{b}}=\frac{64{\text{π}}^{2}}{12}\frac{E}{1-{\text{ν}}^{2}}\frac{t_{\text{PM}}^{3}}{A_{\text{ring}}^{2}}l_{\text{chamber}}$$

with *E* being the apparent elastic modulus of fresh (i.e. never-dried) pit membranes of the current year, *ν* eing the Poisson ratio (here 0.5), *t_PM_* eing the thickness of fresh pit membranes of the current year, *A*_ring_ eing the effective membrane area that was not covered by the projection of the outer pit aperture, and *l_chamber_* eing the chamber depth.

## Results

### Morphology of intervessel pit membranes in earlywood of *Clematis vitalba*

**Figure 2:**
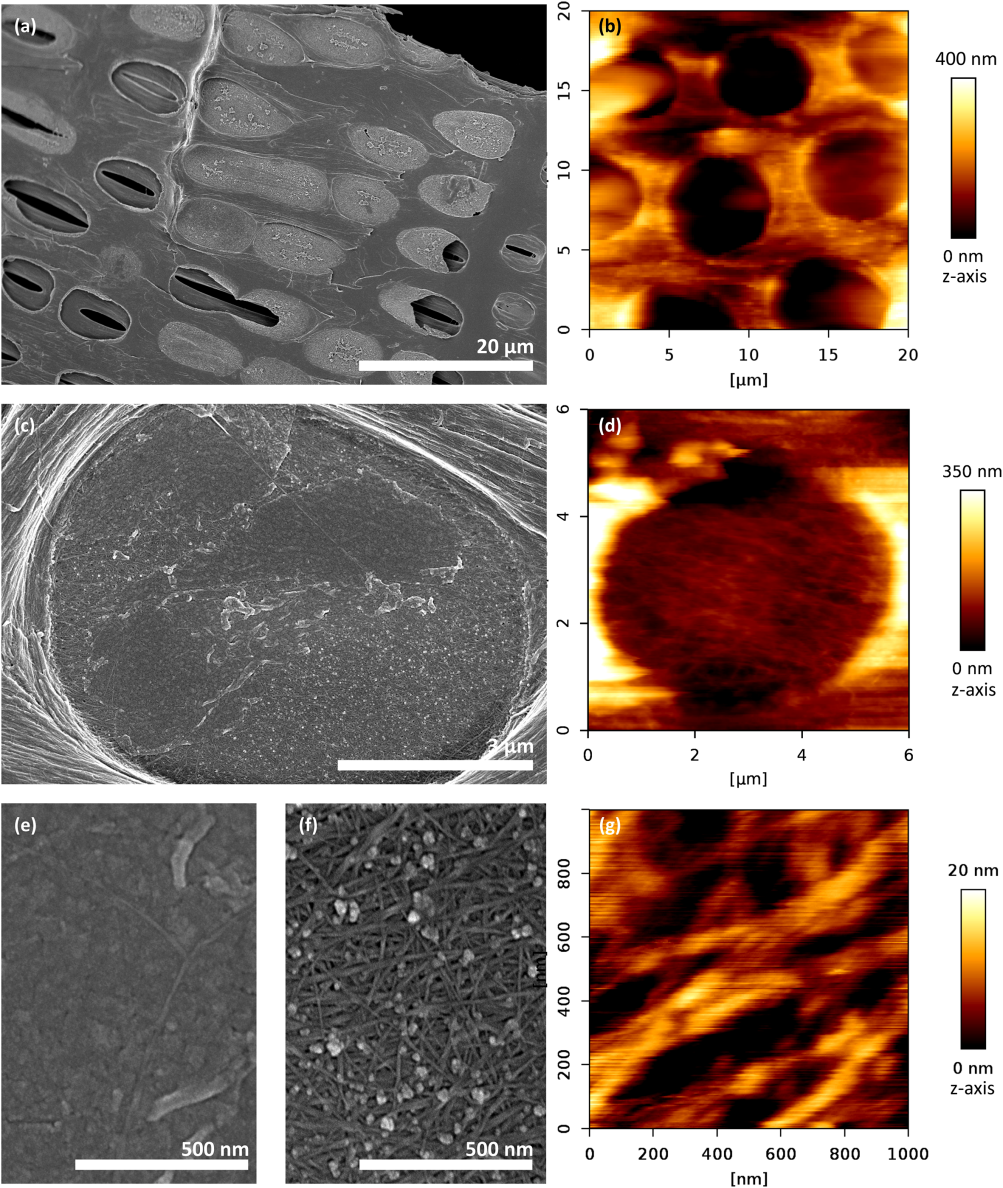
Exposed intervessel pit membranes of *Clematis vitalba* after removal of the overlying secondary cell wall. Left column: SEM, right column: AFM, Tapping mode. **(A, B)** Overview: Pit membranes were either intact (i.e., covering the entire pit border), incomplete (i.e., partly broken), or completely removed during sample preparation. **(C, D)** Single pit membrane, format-filling. **(D, G)** Cellulose microfibrillar aggregates make up the main component of the pit membrane. **(C, E, F)** The membrane is partly covered with a non-cellulosic layer, or at least associated with micelle-like particles.

Intervessel pit membranes imaged using SEM and AFM were intact (i.e., with a pit membrane covering the entire pit border) or incomplete and damaged during sample preparation (**Figures 2A, B**). **Figures 2B, C** provided an overall view of a pit membrane, which had a non-homogenous structure due to local deposits (**Figures 2E, F**), and a fibrous appearance (**Figure 2G**). In many cases, a non-cellulosic coating could be seen (**Figure 2E**), while individual cellulose microfibrillar aggregates could clearly be identified in other parts (**Figures 2D, G**). Globular or micelle-like particles, as described in Pesacreta et al. (2005) were associated with the cellulose microfibrillar aggregates, and seemed to be present also underneath the non-cellulose coating (**Figure 2F**). Never-dried pit membranes from the current year observed with AFM showed less tearing and damage than rehydrated pit membranes of the current year. Older growth rings also showed a higher frequency of incomplete pit membranes and/or those from previous years, although this difference was not quantified.

Fresh, hydrated cellulose-microfibril-aggregates were imaged in high resolution using AFM tapping mode (**Figure 2G**). The diameter of cellulose-microfibril aggregates was on average (*n* = 4)


$${\bar{d}}_{\text{cellulose},\text{ AFM}}=89\;\pm \;13\;\text{nm }\left(\text{mean }\pm \text{ SD}\right).$$

Dried cellulose-microfibril-aggregates were imaged in high resolution using a Hitachi SEM tapping mode (see for example **Figure 2F**). The diameter of the cellulose-microfibril aggregates under SEM were on average (*n* = 4)


$${\bar{d}}_{\text{cellulos},\text{SEM}}\;=11.2\;\pm \;1.9\;\text{nm }\left(\text{mean }\pm \text{ SD}\right).$$

The diameter of fresh, hydrated, pit membranes developed in the current year was imaged using AFM QI mode. The diameters were normally distributed (*n* = 27, Shapiro Wilk: P = 0.417) and had an average diameter


$${\bar{d}}_{\text{PM}}=6.5\;\pm \;1.0\;\text{µm }\left(\text{mean }\pm \text{ SD}\right).$$

Measurements of *d*_PM_ ere also made based on SEM images, disregarding the age of the growth ring. In addition, we measured the outer aperture diameter.


$$d_{\text{aperture}}\;$$

to calculate the area *A*_ring_ ()f the pit membrane that would be supported by the pit border in case of full aspiration. Again, *d_pm_* as normally distributed (*n* = 89; Shapiro Wilk: P_PM_ = 0.134), while


$$d_{\text{aperture}}\;$$

was not (*n* = 89; Shapiro Wilk: P_aperture_ = 0.006), and values were:


$${\bar{d}}_{\text{PM}}=6.9\;µ\text{m }\pm 0.8\;\text{µm }\left(\text{mean }\pm \text{ SD}\right).$$

$${\bar{d}}_{\text{aperture}}=3.1\;µ\text{m }\pm 0.7\;\text{µm }\left(\text{mean}\pm \text{SD}\right)$$

**Figure 3:**
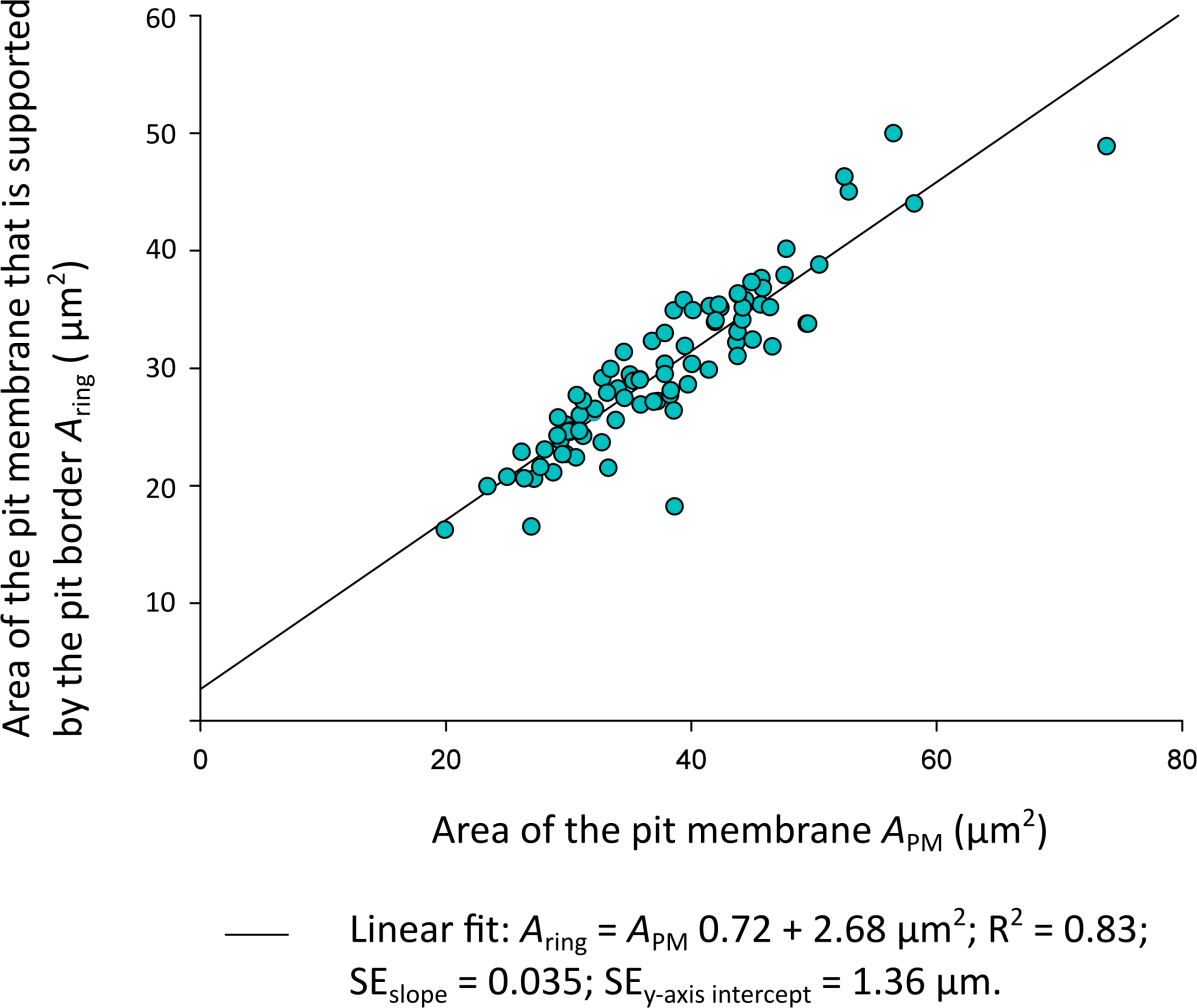
Plot of the *Clematis vitalba* pit membrane area *A*_ring_ (µm^2^) supported by the pit border against the total pit membrane area *A*_PM_ (µm^2^). Linear regressions: f (*x*) = 0.72*x* + 2.68; R^2^ = 0.83. SE_slope_=0.035; SE_y-axis intercept_ = 1.36 µm.

*A*_ring_ was not normally distributed (*n* = 89; Shapiro Wilk: P(*A*_ring_) = 0.01), so a correlation analysis with Spearman’s Test for non-normally distributed data was applied to analyse *A*_ring_ and *A*_PM_. *A*_ring_ orrelated significantly and positively with the total membrane area *A*_PM_ ()**Figure 3**; Spearman’s Test: Correlation coefficient ρ = 0.909; P = 0.0000002). Spearman’s ρ =1 corresponded to a correlation of 100%. Simplified to a two-dimensional view, the pit membrane area supported by a pit border in *Clematis vitalba* was thus 90.9% defined by the total pit membrane size.

**Figure 4:**
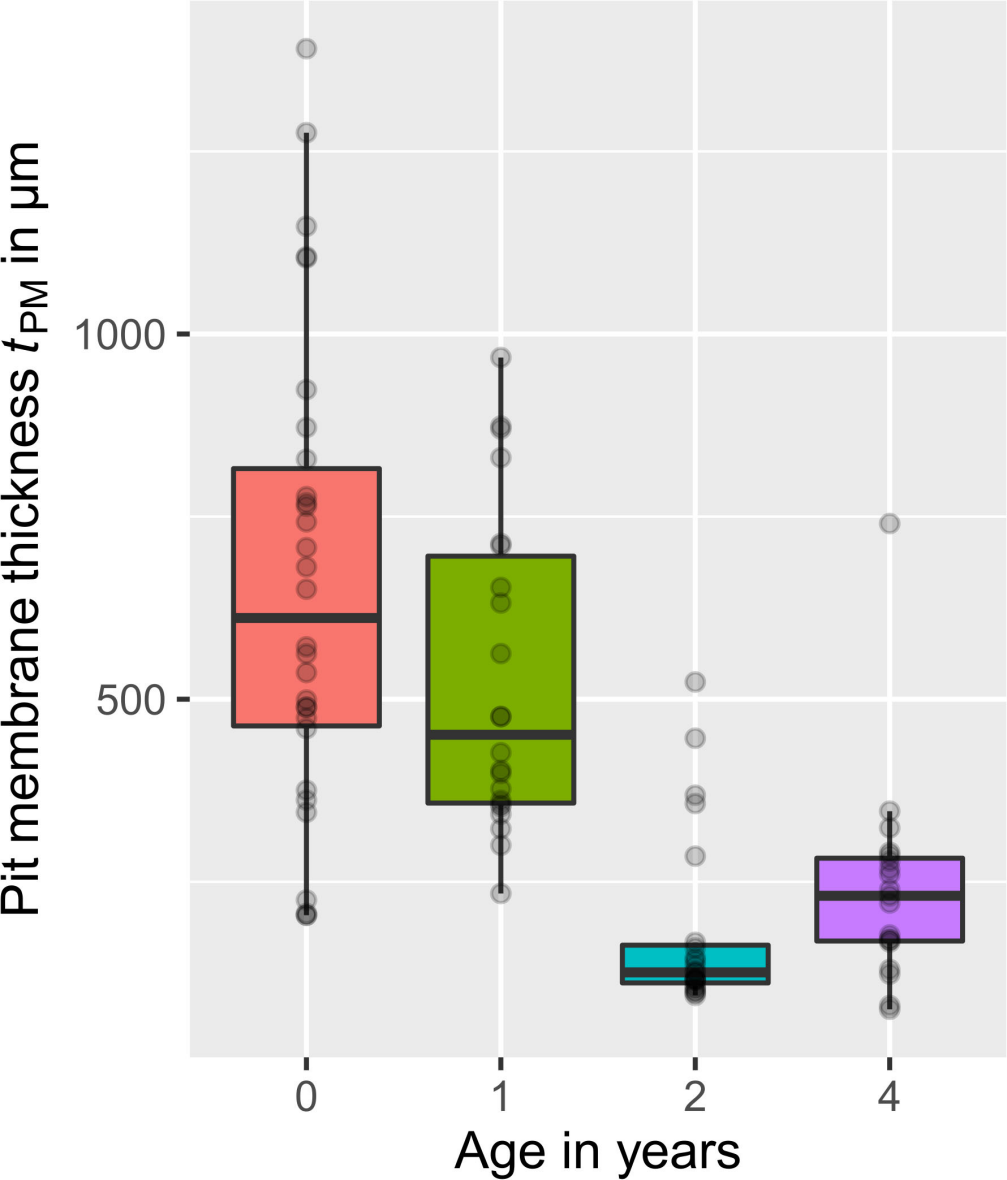
Thickness of *Clematis vitalba* intervessel pit membranes for different ages, visualised in boxplots. Age = 0 years (**≙** current year, *n* = 30), 1 year (*n* = 22), 2 years (*n* = 23), and 4 years (*n* = 19). Grey dots: data points. Pit membranes from the current year showed the highest thickness. Membranes that experienced one winter season were thinner. With further winters, the decreasing trend becomes more pronounced, even if in the fourth year the thickness is again slightly higher than the third year.

The pit membrane thickness *t*_PM_ of three *Clematis vitalba* individuals was measured for four different growth-ring ages (age = 0 (≙ current year), 1, 2, 4 a) based on TEM samples and represented in **Figure 4**. The data were not normally distributed (*n* = 94; Shapiro Wilk: P_thickness_< 0.001). The median thickness of pit membranes developed in the current year was 611 nm (range: 204 – 1391 nm, *n* = 30). Older pit membranes that were one, two and four years old showed a thickness of 451 nm (range: 234 – 968 nm, *n* = 22), 126 nm (range: 95– 524 nm, *n* = 23), and 231 nm (range: 76 – 741 nm, *n* = 19), respectively. A significant difference in the age – pit membrane thickness relationship was only found (Kruskal-Wallis One Way Analysis, P_<_ 0.001). The results highlighted that the thickness of the pit membranes shrunk to less than half in vessels that were more than two years old. As we sampled in different years (2019 and 2021), we obtained from the same individual plant thickness data for age=0 in 2019 (*n* = 3) and age = 2 in 2021 (*n* =13). For age 0, the mean thickness was 806 nm, and 231 nm for age 2.

A Shapiro Wilk analysis for the chamber depth (*l*_chamber_ *)* revealed that the data were normal distributed (*n* = 25; Shapiro Wilk: P_chamber_ = 0.5444). The average chamber depth was *l_chamber_* = 1254 ± 90 nm (MV ± SD).

The ratio between the depth of the pit chamber to the radius of the pit membrane (*l:r*) was 0.36, which was based on the estimated values of ${\bar{d}}_{\text{PM}}\;$ resh, never dried pit membranes) and _mber_.

### Mechanical properties of intervessel pit membranes of *Clematis vitalba*

**Figure 5:**
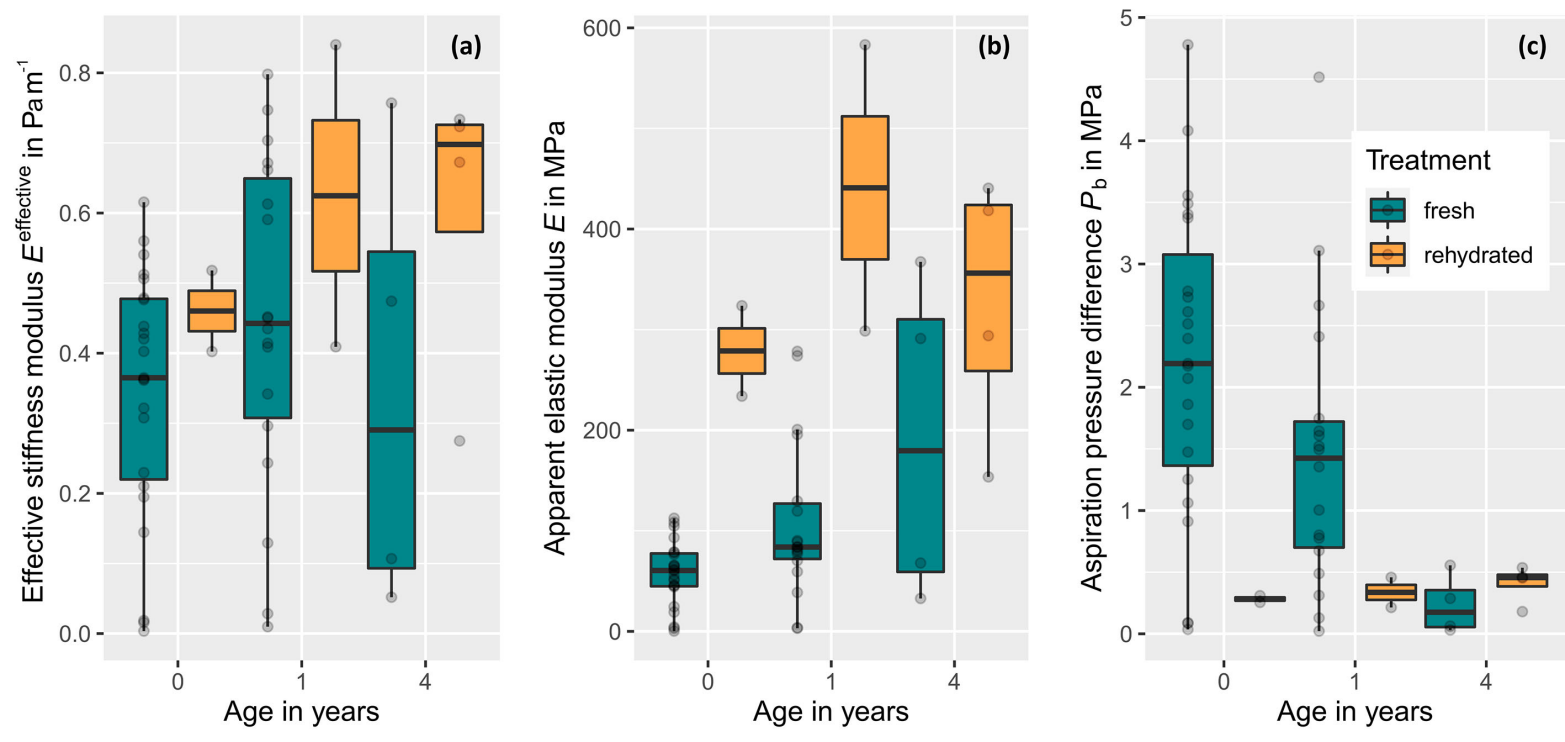
Stiffness parameters and aspiration pressure of intervessel pit membranes of Clematis vitalba for xylem with a different age and hydration status (fresh, never-dried pit membranes: age = 0 years (≙ current year, n = 23), 1 year (n = 18), and 4 years (n = 4) and dehydrated-rehydrated ones: age = 0 years (≙ current year, n = 2), 1 year (n = 2), and 4 years (n = 4).). **(A)** Effective stiffness modulus Eeffective,which is the only from the three parameters represented here, that is directly measured. **(B)** Apparent elastic modulus Eapparent, which represents a transformation of Eeffective by size and thickness t and thus is dependent on Eeffective. Differences in the pattern between both moduli are caused by size and thickness integration. For fresh membranes, thickness data were measured. For rehydrated ones, we used the value of fresh pit membranes with age = 4, assuming the thickness would be similar. Therefore, all dehydrated values in **(B)** are an approximation and do not represent valid, measured data. **(C)** Aspiration pressure Pb, which represents a rough estaimation based on Eapparent, membrane thickness t, and chamber depth l. For fresh pit membranes, Pb decreased significantly with age.

We tested age, hydration status, and size of intervessel pit membranes for their relation to pit membrane mechanics. As older and rehydrated membranes were easily damaged/broken, our dataset remained rather small. Statistically robust data could only be collected for years 0 and 1 for hydrated pit membranes (*n*_age=0, hydrated_ = 23, *n*_age=1, hydrated_= 18), which were described here. For the sake of transparency, however, we included all data in **Appendix S 4** and visualised our results in **Figure 5**.

The apparent elastic modulus (*E* ^apparent^) vs the total pit membrane area (*A*_PM_) interrelation was tested for fresh pit membranes of age = 0 to check the membrane model for quality. *E* ^apparent^ should be independent of pit membrane size as it was an intrinsic material property. Indeed, *E* ^apparent^ showed no correlation with membrane area, which was also confirmed by a linear model (R^2 =^ 0.1348), confirming that the elastic modulus was independent of membrane size. This in turn confirmed the validity of the approach used.

The effective stiffness modulus (*E* ^effective^) represented a mechanical property of a pit membrane as a unit, can be obtained from the measurements without prior knowledge of the material thickness and size, and allows statements about the forces to deform the target. These values were mapped for the entire grid image of each pit membrane measured (**Appendix S 3J**). The effective stiffness for hydrated pit membranes increased with age for age = 1 compared to age = 0 (**Figure 5**), but was even lower in the 4^th^ year than in the current year. However, this was just a trend as the results were not significant (Kruskal-Wallis chi-squared = 1.8923, df = 2, p-value = 0.3882). For rehydrated pit membranes, *E* ^effective^ seemed to be consistently higher than fresh pit membranes. Due to the small sample size, however, no statistically valid statement can be made here. Thus, we observed a trend in mechanical properties, i.e. increased effective stiffness in dehydrated/rehydrates compared to fresh ones, but not in fresh pit membranes with older age compared to younger ones.

Modifying *E* ^effective^ according to Equ. 5 by including size and thickness data revealed the apparent elastic modulus *E* ^apparent^, which represents a material property of pit membrane material. Contrary to *E* ^effective^, an age-dependent continuous trend was found for fresh membranes: *E* increased continuously from age 0 to 4. A Kruskal-Wallis Test with pairwise Wilcox test showed significant differences in pit *E* between age 0 and 1 (Kruskal-Wallis chi-squared = 7.3517, df = 2, P = 0.02533).

As being dependend on *E* ^effective^, a trend in increased *E* ^apparent^ for rehydrated membranes with age was found (**Figure 5**). However, these values are based on the assumptions that dehydrated pit membranes and fresh 4 year old membranes are about the same thickness and must be regarded with particular care.

For fresh pit membranes of the current year, the aspiration pressure needed to deflect a membrane to the pit border (*P*_b_) was 2.20 ±28 MPa, and then reduced to 1.46 MPa after one season. In the 4^th^ year, the smallest value of *P*_b_ was 0.23  ±  24 MPa (**Figure 5**). A Kruskal-Wallis Test with pairwise Wilcox test confirmed a significant decreasing aspiration pressure of the pit membrane with age (Kruskal-Wallis chi-squared = 10.581, df = 2, P = 0.005039).

### MRI observations of stems of *Clematis vitalba*

**Figure 6:**
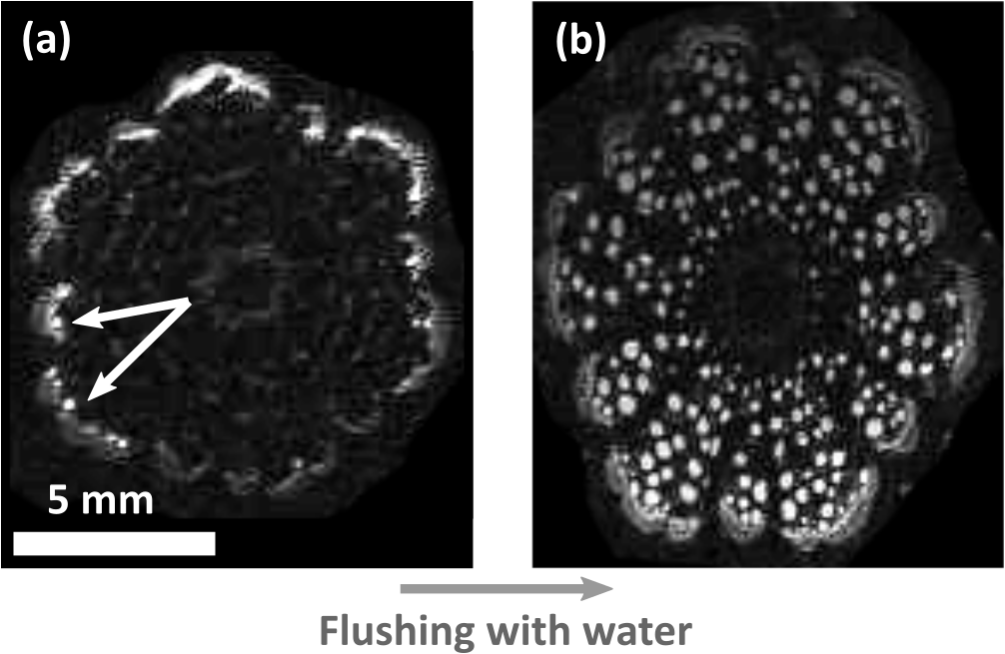
Magnetic resonance images of *Clematis vitalba* stem. **(A)** Living plant. The functional conductive xylem area is restricted to the outermost growth ring (white conduits, indicated by white arrows). Only a few active vessels were detected. **(B)** Flushed cross section. Conduits of all growth rings are water-filled after flushing. The images were equally contrasted by image analysis to highlight the functional conduits.

MRI scans illustrated the hydration pattern in *Clematis vitalba* stems as shown in **Figure 6**. While only some sap-filled and therefore active vessels were detected in the outermost growth ring of an intact plant (**Figure 6A**), the same stem segment showed water-filled conduits across the entire stem section after flushing the sample with water to refill embolised, non-functional vessels (**Figure 6B**). These observations showed that the MRI resolution was high enough to distinguish water-filled vessels from embolised vessels. Moreover, this experiment also showed that the conductive xylem area was restricted to the outermost growth ring and that *Clematis vitalba* was not able to refill its embolised vessels.

## Discussion

Our measurements in the context of membrane stiffness of never-dried intervessel pit membranes of a common European liana did not show a size effect, but an ageing effect in thickness, revealing considerable shrinkage of intervessel pit membranes across growth rings. The decreasing thickness may lead to an ageing effect in the calculated material stiffness (apparent elastic modulus *E* ^apparent^) and the aspiration pressure, which showed a statistically significant decrease with increasing age. As far as we know, these data represent the first estimations for fresh angiosperm pit membranes in their native state.

Valid measurements of rehydrated samples were not successful, but at least indicate an irreversible shrinkage effect, which is largely in line with the irreversible shrinkage of pit membranes during dehydration (Zhang et al., 2017; Jansen et al., 2018; Zhang et al., 2020; Kotowska et al., 2020). The results obtained are discussed below in the context of methodology and their biological meaning.

### Comparison of our mechanical pit membrane approach with earlier work

Following up earlier work by Pesacreta et al. (2005); Capron et al. (2014), and Tixier et al. (2014), we applied an experimental approach to analyse pit membrane mechanics using an AFM indentation method. This approach complements numerical micromechanical methods and theoretical calculations based on the mechanical properties and geometric dimensions of the individual cellulose fibrillar aggregates in pit membranes (Sperry and Hacke, 2004; Li et al., 2020). Our experimental method provided several advantages over purely theoretical approaches. First, as we performed measurements in mineral water with controlled ion composition, we assumed that the pit membranes measured were in a native to close-to-native state with respect to hydration state, and that their properties corresponded to those of a pit membrane *in planta* at night i.e., in a relaxed state without sap flow (Choat et al., 2004). Thus, the pit membranes observed included cellulose fibrillar aggregates that do not experience any strain, except for the innermost, early formed cellulose layers, which may experience a growth stress during cell expansion (Schmid and Machado, 1968; Choat et al., 2008). Interestingly, we encountered non-cellulosic substances/particles on pit membranes, which are likely to represent micelles of polar lipids, and potentially also proteins (Schenk et al., 2018; Schenk et al., 2021; Guan et al., 2022). The presence of micelles may explain the granular structures observed in our SEM images (**Figure 1F**). AFM observations of xylem sap lipids at equilibrium surface tension showed that these form multiples of 4 nm, which corresponds to the height of a fully hydrated lipid bilayer (Yang et al., 2020). Probably, their effect on the stiffness measurements is low, as the indentation depth (more than 100 nm) was several times higher than the particle size.

Secondly, our observations do not rely on the geometric dimensions of individual cellulose fibrillar aggregates, which in turn may depend on the hydration state, and affect the applied imaging technique. We measured higher values of fibrillar aggregate diameters when using AFM on hydrated samples in comparison to SEM observation of dried samples. Overall, the AFM values of the fibrillar aggregate diameters were with ca. 90 nm substantially higher than previously published values of 20-30 nm (Kaack et al., 2019), 20.17 nm (Jansen et al., 2009), and 25-50 nm (Pesacreta et al., 2005). Some uncertainty may come from the t finite width of the cantilever. This overestimation of the cellulose fibre aggregates is particularly important when the material that is scanned and the AFM tip have more or less similar dimensions (Hanley et al., 1992; Pesacreta et al., 2005). The measurements of the effective stiffness modulus *E* ^effective^ are performed with spheres of a radius of 500 nm, an order of magnitude larger than the fibre diameter. Hence, during our measurements, we did not bend an individual fibre, but averaged over a larger area and measured the bending of the composite membrane.

To deduce apparent elastic modulus (material stiffness) values from experimental indentation data, we applied a membrane model that is appropriate for analysing the centre of a thin plate with clamped edges. The quality of our model is strengthened by the finding that the apparent elastic modulus was independent of the membrane size. This behaviour was expected since (1) the structural concept of a homogenous pit membranes applies theoretically to the entire central area and is independent of membrane size, and (2) the apparent elastic modulus is a material constant and independent of dimensions. Nevertheless, a missing negative apparent elastic modulus – total pit membrane area interrelation does not indicate that the absolute elastic values are automatically true. Regardless of the accuracy of our absolute values, the relative elasticity differences represent meaningful information with functional significance.

### Does the elasticity of pit membranes change with age *in Clematis vitalba?*

Studies on the variation of pit membrane features within an individual species are rather sparse (Kotowska et al., 2020). We contributed to filling this gap by analysing stiffness moduli of intervessel pit membranes of *Clematis vitalba* across several growth rings. Most importantly, values of the effective stiffness modulus (*E* ^effective^) and apparent elastic modulus (*E* ^apparent^) of fresh pit membranes increased to about 150% and 130%, respectively, after one winter (**Figure 5**), while the aspiration pressure (**Figure 5**) decreased.

An important difference noted between *E* ^apparent^ and *E* ^effective^ was for fresh pit membranes of age = 4 years. While values of *E* ^effective^ were lower than those obtained for current year pit membranes, values of *E* ^apparent^ increased to 316% of the initial value measured for current year pit membranes. This difference might show the influence of the membrane thickness on its elasticity: Consideration of the units of the parameter *E* ^effective^ $\left(\frac{\text{Pa}}{\text{m}}=\frac{N}{{\text{m}}^{3}}\right)\;$ th N being Newton, suggests that the parameter should be interpreted as a force that is needed to deform a target and thus should be taken as a mechanical property of a pit membrane as a discreet object, while *E* ^apparent^ is a mechanical or material property of the stuff the pit membrane is made out of. Although older pit membranes tend to have a higher material stiffness than current year ones (compare age = 4 and 0 in **Figure 5B**), they seem to be easier to deflect than younger membranes, which are softer but thicker (compare age =4 and 0 in **Figure 5A**). With exception of the one-year old membranes, the behaviour of fresh membranes as a unit (*E* ^effective^) is in line with the behaviour of the age-related aspiration pressure values *P*_b_ (**Figure 5C**). This estimated aspiration pressure of the pit membrane shows a clearly and significant age-related decrease with increasing age.

One may wonder whether *E* ^effective^ represents the most useful parameter out of the three mechanical ones. On the one hand, unlike the other parameters, it is directly measurable; on the other hand, it represents biological relevance and helps to estimate relative membrane behaviour.

### Do pit membranes shrink over years in *Clematis vitalba* and what we can learn from this?

Comparing the trends in **Figures 4**, **5** we can deduce that the pit membrane thickness is particularly important for mechanical properties of pit membranes. We found a significant trend of thickness decreasing with age. This trend we interpret as shrinkage effect and not as age effect, which would mean, that juvenile plants would produce thinner pit membranes than older plants. There is available evidence for pit membrane thickness of intervessel pit membranes being largely similar in xylem of young branches, leaves, mature stem wood, and roots, and independent of the age of the plant. Differences in pit membranes thickness across organs have been found, for instance, in *Acer pseudoplatanus* (Kotowska et al., 2020), but these differences are much smaller than those caused by a gradual shrinkage process over time. Also, we have shown in another study that pit membranes thickness is largely unrelated to conduit dimensions (Lens et al., 2022). There is also published evidence that pit membranes in *Vitis vinifera* plants cultivated in Israel undergo a 50% shrinkage within a single growing season (Sorek et al., 2021). Additionally, our thickness results on the same individual measured in two different years, supported a shrinkage/aging effect compared to an age effect.

There is convincing evidence that irreversible shrinkage of pit membranes is caused by dehydration, which induces the formation of strong hydrogen bonds between cellulose components, making them more compact (Zhang et al., 2017; Zhang et al., 2020; Kotowska et al., 2020). The shrinkage observed in *Clematis* over several growth rings is in line with a similar pit membrane shrinkage reported in grapevine, but within a single growing season (Sorek et al., 2021), and confirms earlier descriptions based on TEM observations of pit membranes by Schmid and Machado (1968). These earlier studies also showed that this shrinkage due to ageing is typically associated with an increase in electron density, most likely due to lipid deposition. The electron density of pit membranes, however, was not measured in this study.

Unfortunately, we were only able to get results for a few rehydrated xylem samples, as a very large proportions of the pit membranes became broken and torn during the dehydration-rehydration process. It is unclear if this tearing represents an artefact or may also occur under natural conditions in the field. This observation is in line with earlier observations by Shane et al. (2000), who described that partial drying of membranes in Maize was sufficient to prompt tearing.

There are two reasons why we can assume that compared to fresh membranes of age = 0, the mechanics of rehydrated membranes of the current year and the mechanics of fresh membranes that experienced several winters are modified in a similar way. First, MRI scans revealed no sap-transporting vessels that are more than one year old, indicating that these are embolised. This finding is directly in line with a staining experiment on *Clematis vitalba* by Kucĕra and Bosshard (1981), and corresponds to the age-dependent embolism pattern in wide earlywood vessels of ring-porous species (Sperry et al., 1994). However, our samples dried out much more during dehydration (23% RH) than they would do *in planta* (fibre saturation point at 30% wood water content). Pit membranes cannot shrink arbitrarily and are found to shrink even by 50% within a year *in planta* (Sorek et al., 2021).

Secondly, physiological tissue senescence such as heart wood formation and tylosis formation is not known for this species, unlike for instance grapevine (McElrone et al., 2021). So, the only change in material properties of pit membranes should be a higher density of cellulose-microfibril aggregates.

Thus, the results can be considered as indirect evidence of dehydrated pit membranes being less elastic than fresh ones. Contrary to the speculation of Kucĕra and Bosshard (1981) that the functionality of membranes would be maintained despite dehydration, we interpret the alteration in thickness and apparent stiffness modulus as an irreversible, physical ageing process, which reduces the functional live-span of vessels in secondary xylem in *Clematis vitalba* to one season, although unicellular tracheids in older growth rings could remain functional (Sano et al., 2011; Fanton and Brodersen, 2021). Yet, MRI observations did not allow us to detect the hydrated status of individual tracheids, which are much narrower than the ca. 50 µm resolution that was achieved. Alternatively, it would be useful to observe the ultrastructure of pit membranes between tracheids in growth rings that are older than the current year.

While the primary cause for gas filling of wide vessels is probably frost-induced embolism, and embolism refilling in our study species is unlikely (Hölttä et al., 2002; Sevanto et al., 2012), it is unclear whether or not the limitation of sap flow to the current year’s growth is beneficial or not.

As *E* ^effective^ seems to enable the detection of dehydration-dependent elasticity differences between pit membranes, without the need to obtain additional parameters (thickness, radius), one may question whether or not the AFM indentation technique would be a suitable tool to identify embolism, or the hydraulic lifespan of conduits. However, given the technical challenges associated with AFM, it is not realistic to use AFM as a method to detect embolism. Even TEM observation of pit membranes across various growth rings is not straightforward.

### Functional interpretation of the stiffness parameters, aspiration pressure and pit membrane geometry

For fresh, never-dried pit membranes from the current year, the highly variable result of *E* ^apparent^ = 57 ± MPa was obtained, for rehydrated samples *E* ^apparent^ was 279  ±  MPa. Direct comparisons between our data and those from Capron et al. (2014) (360 MPa) cannot be made due to slightly different methods. Nevertheless, we find fairly similar *E* ^apparent^ values for shrunken pit membranes.

Values of *E* ^apparent^ obtained for *Clematis* is about 2.5 times lower than for *Populus deltoides* x *nigra*, although the membrane thickness of *Populus* was thinner (on average 310 nm) than *Clematis* (on average 610 nm) for vessels from the current growth ring. It is unclear whether our discrepancy is due to systematic error, for example, non-exclusive removal of upper pit membrane layers when the pit border pair is opened up, potentially making the pit membrane thinner than it initially is. Additionally, during sample preparation, a gel-like substance was observed on transverse surfaces after cutting. Such fairly fast wound response has also been described for another *Clematis* cultivar, albeit with a longer time lag (Jedrzejuk et al., 2012), and is a phenomenon frequently described for cut flowers (van Doorn and Cruz, 2000; Loubaud and van Doorn, 2004). In our case, it could have led to a disturbance of the sensitive cantilevers, and thus to a slight falsification of *E* ^effective^ and *E* ^apparent^. Nevertheless, fresh pit membranes that were four years old gave broadly more or less similar *E* ^apparent^ values as Capron et al. (2014), who studied dried pit membranes, and dried-rehydrated ones. This finding may indicate that both methods are compatible and appropriate.

We deduced the aspiration pressure *P*_b_, to understand the biological relevance of the both, *E* ^apparent^ and *E* ^effective^. For fresh pit membranes of the current year, the net pressure needed to deflect a membrane do the border was *P*_b_ =2.20 MPa, reduced to *P*_b_ = 1.46 MPa after one season and to 0.23 MPa in the 4^th^ year. These values for current-year membranes are higher than the modelled aspiration pressure of gymnosperm pit membranes (0.502 kPa; Schulte et al., 2015). Even if the pit membrane pressure that is caused by flow would be much higher in angiosperms due to the absence of a margo, we assume that angiosperm pit membranes would not be deflected by hydraulic resistance only. Comparing *P*_b_ with a realistic water potential of *Clematis vitalba* experienced in the field during summer (ca. -1.5 MPa) (Cochard et al., 2010), may indicate that intervessel pit membranes between conductive and embolised conduits are not aspirated in the field. However, it is unclear if the xylem water potential reported by Cochard et al. (2010) is really a seasonal summer minimum that this species may experience in summer.

The quotient of pit chamber depth and pit membrane diameter (*l:r*) has been suggested to be positively correlated with embolism resistance (Capron et al., 2014; Levionnois). We found the *l:r* value of *Clematis* (0.37) to be slightly higher than for *Fraxinus americana* (0.31; Choat et al., 2004). At the pit level, *Clematis* should therefore be somewhat more susceptible to embolism than *Fraxinus americana*. Moreover, there is experimental evidence for a relationship between embolism resistance and the pit membrane diameter-to-thickness ratio (Levionnois et al., 2021; Levionnois et al., 2022), suggesting that the deflection resistance of pit membranes may affect embolism propagation. Yet, it remains unclear how pit membrane thickness of fully hydrated, non-shrunken pit membranes may affect its elasticity modulus. It has also been suggested that vestured pits, which are characterised by small, frequently branched protuberances that may occur near the outer pit aperture or completely fill up the entire pit border, could increase embolism resistance by providing mechanical support to the pit membrane (Zweypfenning, 1978; Jansen et al., 2004; Medeiros et al., 2019). While pit membranes in species with vestured pits can be thinner than non-vestured pit species (Levionnois et al., 2021; Levionnois et al., 2022), more research is needed to make generalisations and a functional interpretation of embolism resistance (Smith-Martin et al., 2022).

## Conclusion

Our data estimate different mechanical properties of fresh, never-dried pit membranes of an angiosperm species, and indicate that intervessel pit membranes of *Clematis vitalba* undergo an irreversible mechanical ageing process.

Shrinkage of pit membranes seems to make pit membrane material stiffer, while a lower force is needed to deflect shrunken pit membranes than fresh ones. This seemingly contradictory finding highlights the complex role of thickness in mechanical properties. As pit membrane thickness shows a five-fold variation across species, which has consequences for the size of pore constrictions for transport (Kaack et al., 2019; Kaack et al., 2021), we expect that the force to deflect a membrane show species-specific differences, and this should be tested based on multiple species from different plant functional groups. Further work is also needed to determine the accuracy of the not directly measurable parameters Apparent elastic modulus and aspiration pressure, and how deformation of intervessel pit membranes affect fluid transport at the pit membrane level.

## Data availability statement

The raw data supporting the conclusions of this article will be made available by the authors, without undue reservation.

## Author contributions

CC performed experiments, analysed and interpreted data, made the figures and wrote the first draft. FP supervised AFM experiments, programmed code for data extraction and did data extraction, analysed data, and contributed to the manuscript. CC and FP share first authorship. SB programmed code for model fitting, helped to interpret the data, and contributed to the manuscript. VR coordinated the MRI experiments and data acquisition, and contributed to the manuscript. K-EG supervised the AFM work of the project, and contributed to manuscript. SJ supervised the project and contributed to the manuscript. All authors contributed to the article and approved the submitted version.
