# Supplementary material for: Ageing-induced shrinkage of intervessel pit membranes in xylem of Clematis vitalba modifies its mechanical properties as revealed by atomic force microscopy
Source: Front Plant Sci. 2023 Jan 23;14:1002711. doi: 10.3389/fpls.2023.1002711 (PMC9899931; doi:10.3389/fpls.2023.1002711)
Supplement: Supplementary file 1 [file Table_1.docx]

Carmesin et al.—Frontiers in Plant Science 2022—Appendix S1


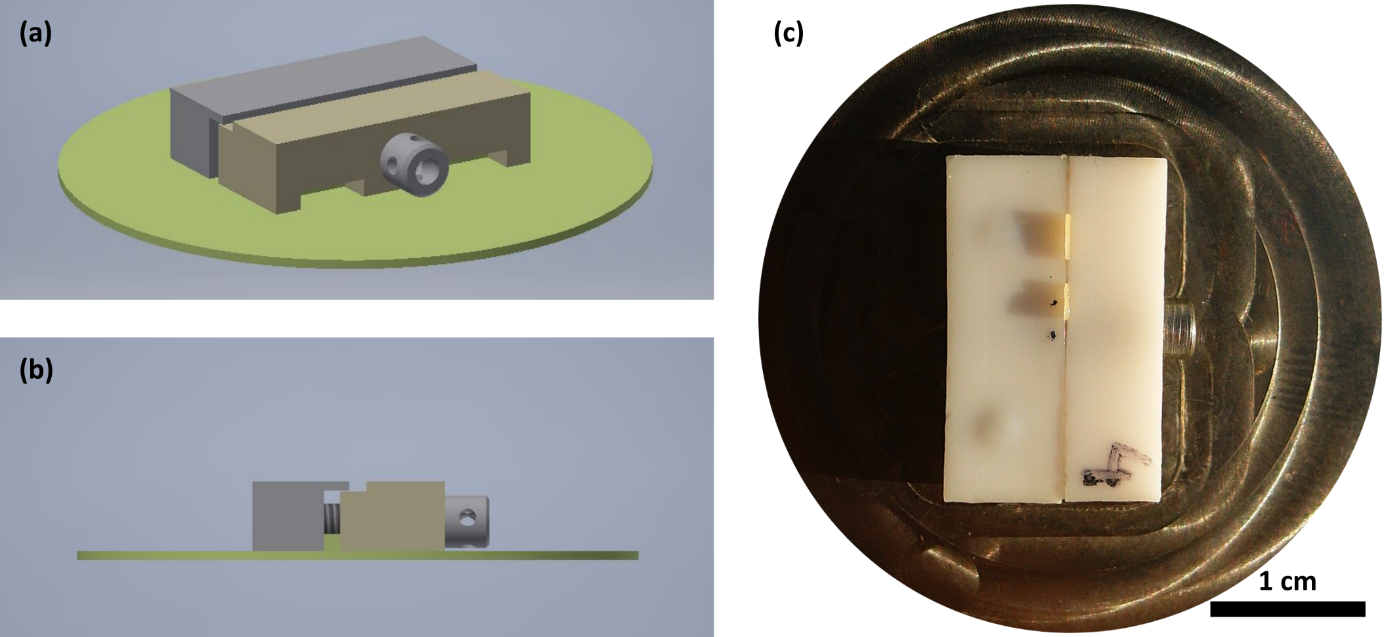


**Appendix 1.** Clamp to fix samples for AFM observation. (a) Front view. (b) Side view (c) Top view. The clamp consists of two parts. The proximal part (light brown in (a) and (b)) can be moved with a screw so that samples of variable thickness (up to ca. 1 mm, (c)) can easily be clamped and detached. The metal base (green in (a) and (b)) provides the required density to keep the samples under water, so these can be scanned when submersed in a liquid without sample movement.
