# Supplementary material for: Ageing-induced shrinkage of intervessel pit membranes in xylem of Clematis vitalba modifies its mechanical properties as revealed by atomic force microscopy
Source: Front Plant Sci. 2023 Jan 23;14:1002711. doi: 10.3389/fpls.2023.1002711 (PMC9899931; doi:10.3389/fpls.2023.1002711)
Supplement: Supplementary file 2 [file Table_2.docx]

Carmesin et al.—Frontiers in Plant Science 2022—Appendix S2

**
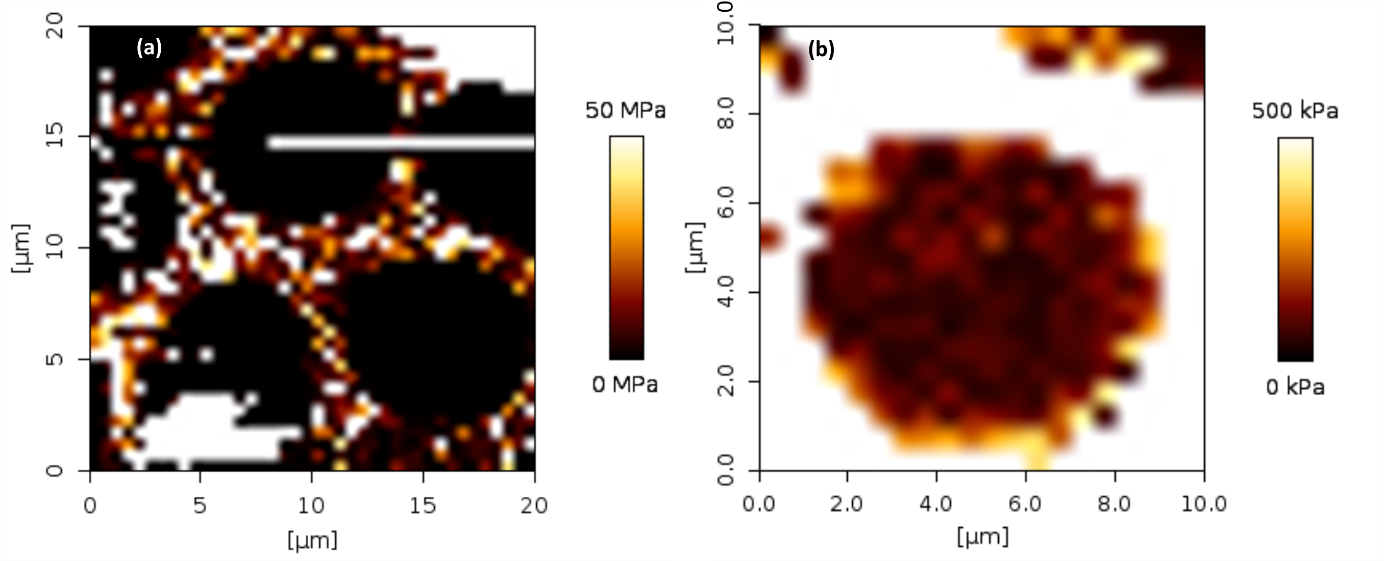
**

**Appendix 2**: Exposed intervessel pit membranes of Clematis vitalba after removal of the overlying secondary cell wall, imaged with atomic force microscopy in QI mode in water. The colours encode an elasticity modulus. However, the underlying model is not suitable for pit membranes. Therefore, this imaging was only used to find pit membranes in the samples. (a) Overview pictures of 20 x 20 µm and 32 x 32 pixels. (b) Detailed picture of 10 x 10 µm and 20 x 20 pixels.
