# Supplementary material for: Ageing-induced shrinkage of intervessel pit membranes in xylem of Clematis vitalba modifies its mechanical properties as revealed by atomic force microscopy
Source: Front Plant Sci. 2023 Jan 23;14:1002711. doi: 10.3389/fpls.2023.1002711 (PMC9899931; doi:10.3389/fpls.2023.1002711)
Supplement: Supplementary file 3 [file Table_3.docx]

Carmesin et al.—Frontiers in Plant Science 2022—Appendix S3

**
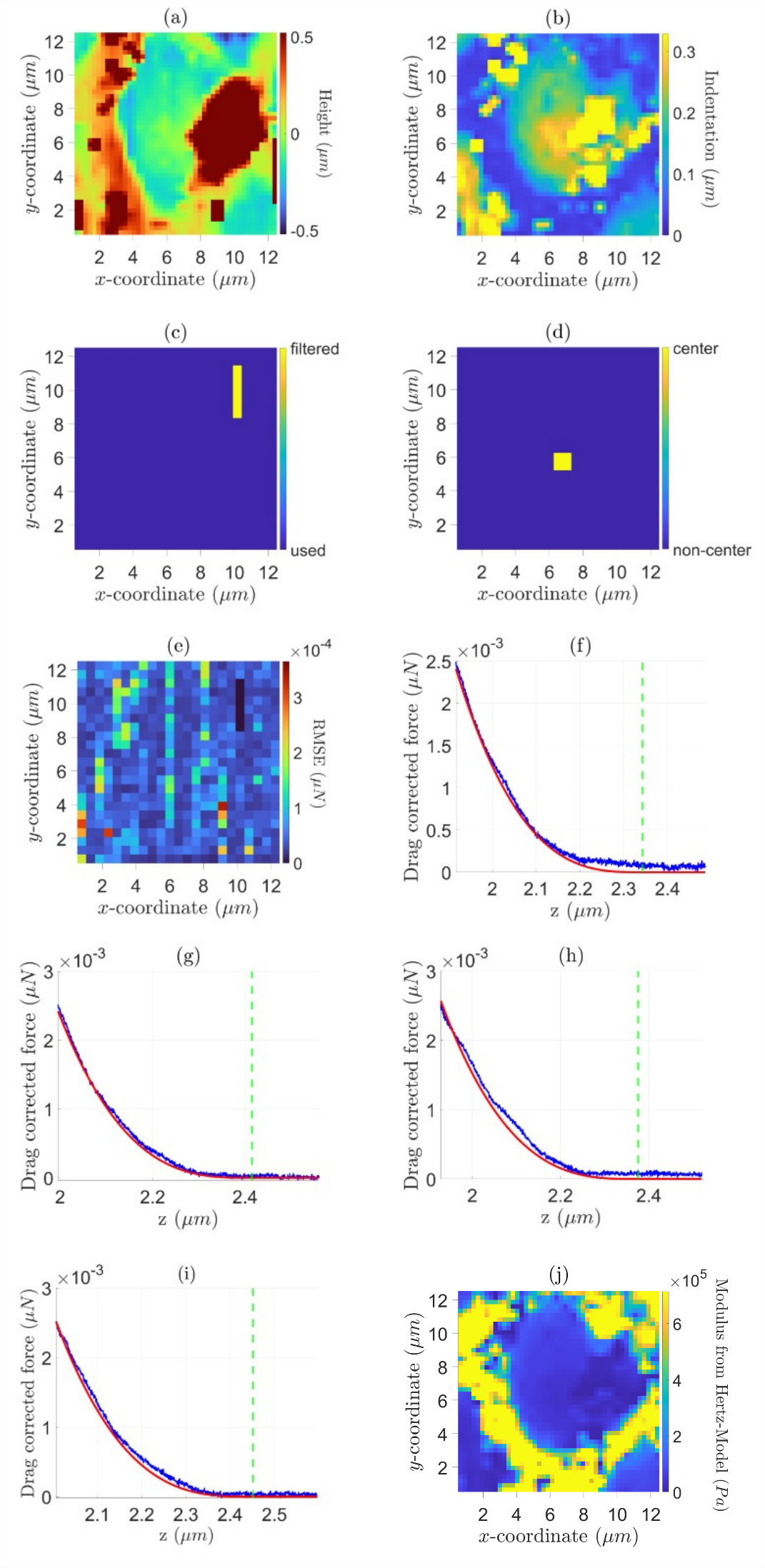
**

**Appendix 3.** Illustration of the procedure to model the Effective stiffness and Apparent elastic modulus of a pit membane. (a) Height profile via Hertz fit. (b) Indentation depth via Hertz fit. (c) Data filtering sorts out curves with less than 700 measurements, penetration events and no/positive slope. (d) Estimated membrane centre based on indentation depth. (e) Goodness of the fit with RMSE < 0.7e10-4. (f)-(i) Force distance curves of the centre, fitted via a non-linear model. (j) Relative stiffness via Hertz fit.
