# Supplementary material for: Ageing-induced shrinkage of intervessel pit membranes in xylem of Clematis vitalba modifies its mechanical properties as revealed by atomic force microscopy
Source: Front Plant Sci. 2023 Jan 23;14:1002711. doi: 10.3389/fpls.2023.1002711 (PMC9899931; doi:10.3389/fpls.2023.1002711)
Supplement: Supplementary file 4 [file Table_4.docx]

Carmesin et al.—Frontiers in Plant Science 2022—Appendix S4

**Appendix 4.** Measured and calculated mechanical parameters for fresh and rehydrated (rehydr.) intervessel pit membranes of Clematis vitalba: Effective stiffness modulus E^effective^, apparent elastic moduls E^apparent^, aspiration pressure P_b._ Green colour: normal distributed values. Red colour: not normal distributed. -: sample size too small for normal distribution test. SD: Standard deviation. SD was used for normal distributed data. For not normal distributed data, the range (min and max) was given.

| **Age** | **Treatment** | **Effective stiffness modulus *E*^effective^** $\boldsymbol{\pm}$**SD or range in N/m^3^** | | **Apparent elastic modulus *E*** $\boldsymbol{\pm}$**SD or range in MPa** | | ***P*_b_ in MPa** | | **Sample size *n*** |
| --- | --- | --- | --- | --- | --- | --- | --- | --- |
|  |  | **Value** | **P_shapiro-wilk_** | **Value** | **P_shapiro-wilk_** | **Value** | **P_shapiro-wilk_** |  |
| 0 | Fresh | 0.34$\pm$0.18 | 0.1375 | 57$\pm$32 | 0.417 | 2.20$\pm$1.28 | 0.7937 | 23 |
| 0 | Rehydr. | 0.46 $\pm$0.08 | - | 279$\pm$64 | - | 0.28$\pm$0.38 | - | 2 |
| 1 | Fresh | 0.44$\pm$0.24 | 0.4451 | 84  (3-278) | 0.01891 | 1.46$\pm$1.15 | 0.1021 | 18 |
| 1 | Rehydr. | 0.63$\pm0.$31 | - | 440$\pm$200 | - | 3.37$\pm$0.17 | - | 2 |
| 4 | Fresh | 0.35$\pm$0.33 | 0.4567 | 190$\pm$165 | 0.3272 | 0.23$\pm$0.24 | 0.4109 | 4 |
| 4 | Rehydr. | 0.70$(0.$28-0.73) | 0.0232 | 330$\pm$130 | 0.4622 | 0.41$\pm$0.16 | 0.1641 | 4 |
